# Distributional health and financial consequences of increased cigarette tax in Iran: extended cost-effectiveness analysis

**DOI:** 10.1186/s13561-021-00328-w

**Published:** 2021-08-13

**Authors:** Behzad Raei, Sara Emamgholipour, Amirhossein Takian, Mehdi Yaseri, Ghahreman Abdoli, Ahad Alizadeh

**Affiliations:** 1 Department of Health Management & Economics, School of Public Health, Tehran University of Medical Sciences, Tehran, Iran; 2 Health Equity Research Centre (HERC), Tehran University of Medical Sciences, Tehran, Iran; 3 Department of Epidemiology and Biostatistics, School of Public Health, Tehran University of Medical Sciences, Tehran, Iran; 4 Faculty of Economics, University of Tehran, Tehran, Iran; 5 Metabolic Diseases Research Center, Research Institute for Prevention of Non-Communicable Diseases, Qazvin University of Medical Sciences, Qazvin, Iran

**Keywords:** Taxes, Elasticity, Cigarette smoking, Outcome assessment

## Abstract

**Background:**

To assess the potential impact of a tax-induced cigarette price increase on financial and health outcomes by different socioeconomic groups.

**Methods:**

In a modeled condition using pooled cross-section data from Household Income and Expenditure Survey (2002–2017) and Iran 2019 population data, a methodology of an extended cost effectiveness analysis (ECEA) was applied to model the impact on cigarette consumption of hypothetically increased cigarette tax. The methodology was employed to evaluate: [1] health benefits (premature deaths averted); [2] health expenditures regarding smoking-related disease treatment averted; [3] additional tax revenues raised; [4] change in household expenditures on cigarettes; and [5] financial risk protection among male Iranian smokers in a time span of 60 years following a one-time increase in cigarette price of 75%. The Stata version 15.1 (StataCorp., College Station, TX, USA) was used to perform the relevant analysis and estimate regression models.

**Results:**

A 75% increase in cigarettes price through taxation would reduce the number of smokers by more than half a million, 11% of them in the poorest quintile; save about 1.9 million years of life (11% of which would be gained in the lowest quintile compared to 20% in the highest one); eliminate a total of US$196.4 million of health expenditures (9% of which would benefit the bottom quintile). Such a policy could raise the additional annual tax revenues by roughly US$ 1 billion, where the top two quintiles bear around 46% of the total tax burden. We estimated that the tax increase would avert an estimated 56,287 cases of catastrophic expenditure that wholly concentrated among the bottom two expenditure quintiles.

**Conclusion:**

Increasing cigarette tax can provide health and financial benefits, and would be pro-poor in terms of health gains, Out-of-Pocket (OOP) savings, and financial risk protection against smoking-related diseases.

**Supplementary Information:**

The online version contains supplementary material available at 10.1186/s13561-021-00328-w.

## Introduction

Tobacco use is extensively acknowledged to be causally linked to developing communicable and noncommunicable diseases (NCDs) [1, 2]. Annually, there were an estimated six million deaths from tobacco-caused diseases and this figure is projected to amount to 8.3 million a year by 2030, with more than 80% of these expected to occur in low- and middle-income countries (LMICs) [3]. By 2030, tobacco use compared to other health risk factors is projected to produce the largest burden of premature morbidity and mortality around the world [4]. WHO estimates of tobacco smoking prevalence in Iran indicate that in 2010 nearly 22% of men and close to 1% of women smoked [5]. According to the Ministry of Health and Medical Education (MOHME), the total number of deaths caused by smoking in Iran in 2013 was almost 70,000. This increased from 50,000 in 2006 and 55,000 in 2008. However, these figures only include those who died as a direct result of smoking and do not include the estimated 140,000 people suffering from chronic tobacco-related diseases [6–8]. By ratifying the WHO Framework Convention on Tobacco Control (FCTC), countries committed to driving prevention initiatives to counteract the globalization of the tobacco epidemic [2]. High taxes comprising 75% or more of the retail price recommended by WHO have been weakly enforced in most low- and middle-income countries such as Iran, implying there is substantial room for taxation as a most effective and efficient instrument for health policy [9–11]. The debate about the impacts of increasing tobacco excise taxes has gained fresh prominence with many arguing that the correct approach must incorporate all of the health, financial, and distributional consequences of raising tobacco taxes together [12–14], including health expenditures saved, tobacco-related premature deaths averted, and change in tobacco tax revenue [15]. Iran has shown poor compliance with the FCTC’s Raise (MPOWER) measure. For example, according to the 2019 WHO report on the global tobacco epidemic, tobacco taxes in Iran remained low, representing only 21.7% of retail prices [9]. According to WHO estimates, Iran will not achieve the smoking component of the global noncommunicable disease target unless effective and sustained action is taken [16]. Among Iranian male cigarette smokers, we quantify the distributional impacts (across expenditure quintiles) of a 75% simulated tax-induced cigarette price increase in terms of: public health benefits (averted premature deaths), health care cost savings (averted treatment cost of tobacco-related diseases), extra tax revenues stemming from increased cigarette tax rates, as well as financial risk protection (averted out-of-pocket and averted cases of impoverishment health expenditures).

## Methods

This paper attempts to quantify the tax burden and health benefits of cigarette excise tax by expenditure quintiles. Since cigarette smoking is more prevalent among males (22% of men are smokers compared to less than 1% of women [17]), our analysis was limited solely to male cigarette smokers aged 15 and above. We applied a methodology of ECEA to estimate the expected cost and consequences of a hypothetically increased cigarette tax, as well as their distribution across different socio-economic subgroups. In a modeled condition using pooled cross-section data from Household Income and Expenditure Survey (HIES) from 2002 to 2017 and Iran 2019 population data, a methodology of ECEA was applied to model the impact on cigarette consumption of hypothetically increased cigarette tax among male Iranian smokers in a time span of 60 years following a one-time increase in cigarette price of 75%. The HIES is administered by the Statistical Center of Iran (SCI) and is representative at the national level. Cigarette demand equations were estimated across 5 income groups (using expenditures as a proxy) independently, allowing us to distinguish between the responses of smokers to price increases. Quintiles were defined by the distribution of households in terms of expenditures per equivalent adult. These quintiles are further divided into 14 age groups.

In the current study, a 2-part estimation procedure was used to model the parameters associated with cigarette smoking behaviors. Conditional and participation price elasticities were estimated for each expenditure quintile separately. We used a two-part model to avoid bias stemming from the censuring of the consumption variable. in addition, the probit model used in part one allows us to estimate the participation elasticity (PE).

### Study population

Using data on the Iranian male population divided into different age groups, we classified the population into 5 expenditure groups which each one was equal in size. The absolute number of current smokers per expenditure quintile was calculated using age-specific and quintile-specific smoking prevalence data (Table 1). Therefore, for each expenditure group, the number of quitters owing to a price increase was the product of (i) the actual number of smokers in that expenditure group, (ii) the participation elasticity of cigarette demand in the same group, and (iii) the actual magnitude of the price increase. Existing literature reveals that apart from adolescent groups, participation elasticity of smoking for different ages is driven mainly or totally by the cessation rather than initiation. Therefore, we assumed that participation elasticity is tantamount to cessation elasticity [32].

**Table 1:** Inputs used for the modeling of the increase in cigarette excise tax in Iran

Input	Value	Source
Male population by age group	• 15–19 y–olds: 2,806,000 • 20–24 y–olds: 2,882,000 • 25–29 y–olds: 3,595,000 • 30–34 y–olds: 4,333,000 • 35–39 y–olds: 4,092,000 • 40–44 y–olds: 3,213,000 • 45–49 y–olds: 2,622,000 • 50–54 y–olds: 2,232,000 • 55–59 y–olds: 1,795,000 • 60–64 y–olds: 1,452,000 • 65–69 y–olds: 990,000 • 70–74 y–olds: 613,000 • 75–79 y–olds: 413,000 • ≥80 y–olds: 524,000	[18]
Cigarette smoking prevalence per expenditure quintile	• quintile I: 0.16 • quintile II: 0.20 • quintile III: 0.21 • quintile IV: 0.21 • quintile V: 0.19	Authors’ calculations based on HIES 2017
Price per pack (20 cigarettes) 2017 US$) commonly smoked in each quintile	• quintile I: 0.85 • quintile II: 0.89 • quintile III: 1.22 • quintile IV: 1.34 • quintile V: 1.15	Authors’ calculations based on HIESs 2001–2017
Proportion of deaths among smokers attributable to smoking	0.50	[19]
Reduction of smoking-attributable death risk by age at quitting	• 15–19 y–olds: 96.9% • 20–24 y–olds: 94.8% • 25–29 y–olds: 92.1% • 30–34 y–olds: 89.2% • 35–39 y–olds: 86.6% • 40–44 y–olds: 83.7% • 45–49 y–olds: 79.5% • 50–54 y–olds: 72.9% • 55–59 y–olds: 62.8% • 60–64 y–olds: 49.9% • 65–69 y–olds: 36.4% • 70–74 y–olds: 24.7% • 75–79 y–olds: 15.7% • ≥80 y–olds: 9.1%	[13]
Proportion of smoking-attributable mortality, by cause	• Heart disease: 0.64 • Lung cancer: 0.06 • COPD: 0.08 • Stroke: 0.22	Global Burden of Disease study [20]
Utilization rates of healthcare services by tobacco-related disease	• Heart disease: 0.73 • Lung cancer: 0.57 • COPD: 0.37 • Stroke: 0.95	[21] and based on [22–24]
Tobacco-related disease treatment costs (2015 US$)	• Heart disease: US$ 1881 • Lung cancer: US$ 1585 • COPD: US$ 627 • Stroke: US$ 270	[25] and based on [26–28]
Relative utilization of health care per expenditure quintile (standardized to Quintile 3 as a reference)	• quintile I: 0.81 • quintile II: 0.92 • quintile III: 1 • quintile IV: 1.01 • quintile V: 1.09	[29]
Annual expenditure per adult equivalent (2017 US$)	• quintile I: 0 to 1664 • quintile II: 1664 to 2426 • quintile III: 2426 to 3361 • quintile IV: 3361 to 5005 • quintile V: > 5005	Authors’ calculations based on HIES 2017
Fraction of healthcare costs paid out-of-pocket	41%	[30]
Cigarette consumption (cigarettes per day) per quintile	Expenditure quintile I to V: {16, 16.4, 16.6, 16.5, 15.7}	Authors calculation from HIESs
Poverty headcount ratio at $3.20 a day (2017, % of the population)	11%	Authors estimation from HIESs, fitted Gamma distribution
Number of male cigarette Smokers per expenditure quintile	• quintile I: 1,041,720 • quintile II: 1,302,300 • quintile III: 1,344,040 • quintile IV: 1,337,630 • quintile V: 1,256,450	Authors calculation based on HIESs
Participation price elasticity of demand for cigarette per quintile	Expenditure quintile I to V: {−0.07, − 0.11, − 0.12, − 0.12, − 0.11}	Authors estimation based on [31] (Supplementary document, **Appendix 3**)

*y* year, *COPD* chronic obstructive pulmonary disease

First, the number of cigarette smokers by expenditure quintile who would stop smoking as a result of increased excise tax was calculated. We do not include health benefits that could be obtained by smokers who reduce consumption without quitting.

### Premature mortality averted

Smoking-related premature death averted by quitting is the primary health outcome. Doll et al. found that smoking accounted for 50% of all deaths among smokers, and therefore reduced the relative risk of premature tobacco-related death is varying, depending on the age at quitting [19]. In this analysis, we used Verguet et al. estimates of the effect on age-specific relative risk reductions [13]. On the assumptions that no current smokers would quit without an increased price policy and half of them die from their addiction over the next 60 years indicating Iranian male life expectancy at age 15 years, we estimated averted deaths using data on prevalence and participation elasticity by the age-quintile group. Furthermore, we estimated (by age group) the cumulative number of years of life gained for quitters following cigarette excise tax increase (75% retail price increase). (Further details were presented in the supplementary document in **Appendix 4** & **5**).

### Financial risk protection

For savings (both private and public health expenditure) in the treatment cost of smoking-related diseases, averted premature deaths were assigned to four main tobacco-related illnesses: heart disease, lung cancer, chronic obstructive pulmonary disease, and stroke. Data on quintile-, and diseases-specific health care utilization rates, as well as average treatment cost per disease, were obtained from the relevant literature and published studies. we adjusted utilization rates relative to the middle quintile as a reference. Owing to the data restriction, we assumed that average costs and smoking-attributable risk are distributed uniformly across all quintiles. We calculated the financial risk protection benefits brought to the households due to the averted out-of-pocket expenditures associated with the treatment of smoking-related diseases, per year. Averted impoverishment is quantified as the number of individuals avoided being pushed under the national poverty line as a result of averted out-of-pocket expenditures linked to smoking-related diseases. In 2017, out-of-pocket health payments as a share of total health expenditure are about 41% in Iran, according to World Bank statistics [30] (supplementary document, **Appendix 8** & **9**).

The issue of thresholds for incidence of catastrophic expenditure due to out-of-pocket health spending has been a controversial and much disputed subject, ranging from 5 to 20% of total household income [33]. In this study, out-of-pocket health expenditure was considered catastrophic whenever it was greater than or equal to 20% of household expenditures per adult equivalent [34]. Catastrophic health expenditure is calculated from national representative data derived from HIESs, based on household expenditures adjusted using the general consumer price index by the year 2017. Averted cases of catastrophic expenditures were calculated as individuals for whom OOP tobacco-related disease treatment costs would have equaled or exceeded 20% of their annual expenditure per adult equivalent.

### Cigarette tax revenues and household expenditure on cigarettes

We calculated post-policy tax revenues generated from raising the cigarette excise tax. The magnitude of annual change is the product of the change in the number of packs of cigarettes consumed per smoker per year, the number of smokers in a given quintile, and the change in excise tax per cigarette pack. Likewise, changes in the amount of household expenditure devoted to cigarettes following increased price were estimated. (More details are given in **Appendix 6** & **7** in the supplementary document).

### Sensitivity analysis

**Fig. 1:**
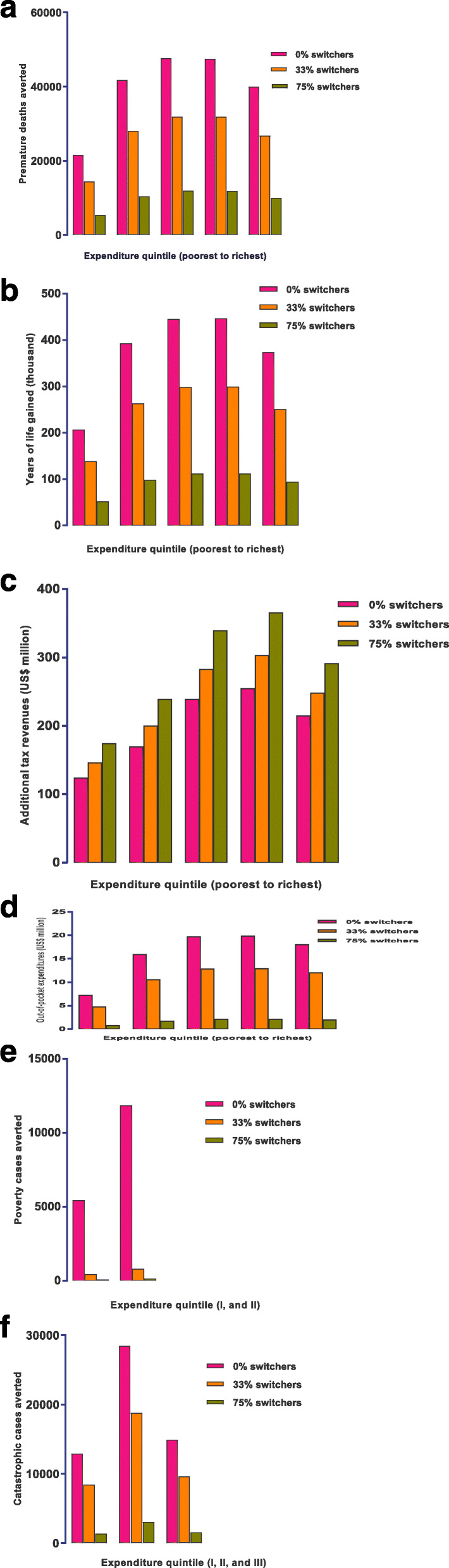
Impact of a 75% increase in the retail price of cigarettes through taxation (percentage of smokers switching to cheaper cigarette brands, i.e., “switchers”, was set at either 0, 33%, or 75%) in Iran, per expenditure quintile, on: the number of smoking–related premature deaths averted (**a**); years of life gained (**b**); the change in annual tax revenues (**c**); the amount of savings in out–of–pocket tobacco–related disease treatment costs (**d**); the number of smoking–related poverty cases averted (**e**); and the number of smoking–related cases of catastrophic expenditures averted(**f**)

To assess the robustness of our findings, we undertook several univariate sensitivity analyses. First, we simulated impact under the scenarios of varying increases in the retail price of a pack of cigarettes (25%- and 100%-point increases). Second, we tested the effect of brand switching by considering a parameter that shows the percentage of smokers who, in response to rising cigarette prices instead of quitting or reducing their consumption, tend to low-priced brands (ie, “switchers”, was set at either 0, 33%, or 75%) on the number of premature smoking-related deaths avoided, changes in tax revenues from cigarettes, years of life gained, and the amount of out-of-pocket expenditures averted by quintiles (Fig. 1). Third, we modeled financial risk protection using two alternative poverty thresholds: a lower poverty line of US$ 1.9, and an upper poverty line of US$ 5.5 per day per headcount (see **Appendix 10** in the Supplementary Document).

## Results

Estimates of participation price elasticities of demand for cigarettes ranged from - 0.07 to − 0.12, and conditional price elasticities from − 0.32 to − 0.40 (Tables 2, and 3 in the supplementary appendix provide more information on regression results). The estimates seem to be consistent with the result of a prior study with an estimate of − 0.45 [35]. The increasing body of research in low and middle-income countries indicates that price affects both participation (prevalence) and consumption, although the magnitude of these effects varies greatly across studies. For example, Jime’nez-Ruiz et al. in Mexico (2007) found that the price elasticity of smoking participation and consumption is − 0.06 and − 0.45, respectively [36]. In contrast, Lance and colleagues (2004) estimated a total price elasticity of − 0.127 with a conditional price elasticity of − 0.026 and participation price elasticity of − 0.101 in Russia [37]. These differences can be accounted for in part by research design and sample sizes.

**Table 2:** Summary findings for the extended cost–effectiveness analysis of the 75% increase in the price of cigarette in Iran, over 60 years

Quintiles	I (poorest)	II	III	IV	V (richest)	Total
Premature deaths averted	21,559 (11%)	41,822 (21%)	47,646 (24%)	47,507 (24%)	39,973 (20%)	198,507
Years of life gained	205,869 (11%)	392,289 (21%)	444,837 (24%)	446,292 (24%)	373,293 (20%)	1,862,580
Expenditures on tobacco-related disease treatment averted (2017; in $US)	17,582,885 (9%)	38,740,848 (20%)	47,973,669 (24%)	48,312,051 (25%)	43,870,212 (22%)	196,479,664
Total OOP savings (2017; in $US)	7,208,983 (9%)	15,883,747 (20%)	19,669,204 (24%)	19,807,941 (25%)	17,986,787 (22%)	80,556,662
Additional tax revenues from excise tax (2017; in $US)	123,410,713 (12%)	169,421,522 (17%)	238,482,157 (24%)	254,234,088 (25%)	214,386,022 (21%)	999,934,501
Change in annual expenditures on cigarette (2017; in $US) % of individual income	33,830,736 (1.95%)	46,443,738 (1.47%)	59,620,539 (1.31%)	57,278,036 (0.85%)	78,318,141 (0.89%)	275,491,189
Poverty cases averted	5418 (0.5%)	11,826 (0.9%)	0	0	0	17,244
No. of cases of catastrophic expenditure averted	12,912 (1.2%)	28,452 (2.2%)	14,922 (1.1%)	0	0	56,287

The distribution of outcomes by percentage per expenditure quintile is represented in parentheses

This study found that a75% price increase in cigarettes would reduce current consumption in all 5 quintiles, pushing 501,735 current male smokers to quit or not to start. This is roughly equivalent to 8% of the total current male cigarette smokers in Iran in 2020. A 75% cigarette price increase in Iran and consequent fall in demand for it would save 198,507 lives and about 1.9 million years of life projected over 60 years, of which 11% would be gained by the lowest expenditure quintile. This distribution is determined by the lower participation price elasticity of − 0.07% for poorer smokers, indicating they are more inelastic with respect to change in cigarette prices, and thus quitting in fewer numbers.

Since the outcomes of interest except for tax revenues occur over long-time horizons in our analysis (60 years), discounting is required to obtain the present value of the stream of two key outcomes including the number of cigarette quitters and years of life gained as a result of the policy implementation. These two key results were presented for a 3% discount rate to illuminate the effect of discounting. When discounting, this policy would avert 134,239 smoking-related premature deaths and would save about 1.1 million years of life. The analysis shows a 75% cigarette tax increase in the following year (end of the first year) would save 6106 lives and 48,727 years of life. Therefore, the average years of life gained per smoker having quitted cigarette smoking is about 4 years or 5.3% of the life expectancy of men in Iran. If “health” is defined by average life expectancy, the health production elasticity of taxing cigarettes on the margin can be estimated using the following equation;

$$ {E}_{Health; Taxation}=\frac{\% change\ in\ Health}{\% change\ in\ cigarrete\  tax}=\frac{\%5.3}{\%75}=0.07 $$

By simple extrapolation, a 1% increase in cigarette tax in Iran (about 1 cent per pack) would mean an increased average life expectancy of 0.07 years for quitters.

We estimated that 85,913 heart disease-related deaths, 8054 lung cancer-related deaths, 10,739 COPD-related deaths, and 29,533 stroke-related deaths could be averted.

A societal perspective was adopted for the analysis and consequences for household private expenditures and health systems were evaluated. From the patient’s viewpoint, we assessed the amount of averted out-of-pocket costs resulting from taxing cigarettes. Depending on the provider’s perspective, the total averted costs of the tobacco-related diseases to the government and social health insurance were estimated. Under the 75% tax, we projected a reduction of US$196.4 million in health expenditures. Consequently, averted out-of-pocket expenditures due to smoking cessation that leads in turn to the lower incidence of smoking-related diseases would aggregate to US$ 80.5 million. Assuming that there is no difference in the distribution of out-of-pocket payments among quintiles, around 22% of the overall out-of-pocket expenditures averted were concentrated in the highest quintile, while the lowest quintile could save 9% of them. Scaled to the total number of male smokers, these figures represent $31 of health care cost savings per smoker, or roughly $13 of OOP savings per smoker.

Based on our estimate, raising the cigarette tax by 75% of retail prices in Iran would generate a total of about US$ 1 billion in excise revenue for 2017, 29% of which would be borne by the bottom two quintiles, compared with 46% in the top two quintiles. In terms of change in expenditures on cigarettes under the same tax increase, we estimated that the lowest quintile would experience 1.95% (US$ 38 million) increases in expenditures while the two highest quintiles would see increases in expenditures on the cigarette of 0.85% (about US$ 57 million) and 0.89% (US$ 78 million) of their budget, respectively. We consider that gamma distribution appreciably fits the data of expenditure as a proxy for income [38], under the fitted model 11% of the Iranian population is already below the poverty line with respect to poverty headcount ratio at $3.20 a day as defined by the World Bank. The number of averted cases of impoverishment was calculated as individuals whose annual income would have decreased to less than US$ 3.20 per day after paying out of pocket for smoking-related disease treatment. In terms of financial risk protection, we estimated that 17,244 new poverty cases and 56,287 cases of catastrophic health expenditures would be averted following a reduction in health expenses resulting from the implementation of the tax policy.

## Discussion

This is the first study in Iran conducted by employing a method of ECEA to quantify the effect of a 75% price increase in cigarettes as proposed by WHO on health benefits, health expenditures, additional tax revenues, changes in household expenditures on the cigarette, and financial risk protection. Depending on the values of included parameters, the results of this study indicate that the policy could avert 198,507 premature deaths from the four major smoking-related diseases and could save about 1.9 million years of life (11% of these in the poorest group) over a 60-year time horizon. and generate cigarette excise revenue amounting to about $US1billion (0.23% of GDP in 2017) in the male population. Increasing tobacco-product taxes will generate sustainable revenues over the short to medium term. In the long run, persistent increases in tobacco tax along with other tobacco control efforts will lead to a higher drop in tobacco consumption and, to a reduction in tobacco taxes revenues. Nevertheless, it is worth noting that decreased tobacco tax revenues would have been replaced by improving public health and averting the costs of the harmful effects of smoking [39].

A rather increasing gradient from the bottom to the top quintiles in the number of life-years gained and in the amount of averted health expenditure on smoking-related diseases might be accounted for the lower prevalence of cigarette smoking, lower participation elasticity, and lower absolute number of smokers among the poorer quintiles. In this study, the impact of the tax policy was found to offer financial protection for households by preventing the risk of impoverishment and catastrophic health expenditure resulted from tobacco-related diseases. The study found that financial risk protection benefits wholly concentrated in the bottom quintiles.

Although the benefits of taxing cigarettes concerning reductions in health care costs and, in turn, the amount of out-of-pocket expenditure, as well as health benefits including premature deaths averted and life-years gained are mainly concentrated among the top 60% of the population, the benefits of financial risk protection would accrue to the bottom two expenditure quintiles. A comprehensive account of impacts does not demonstrate that cigarette taxation to be regressive, especially if the price elasticity of demand for tobacco products and price increases would be large [40]. As Table 2 shows, cigarette taxation would have a greater impact on richer groups. Although these results differ from some published studies [12, 15, 41], as to which strata of society benefit, they are consistent with findings of a study that has examined the effect of the tobacco tax in the Kyrgyz Republic [42]. The study has confirmed the findings of a similar previous study in Turkey in 2010 which found cigarette price increase would reduce the number of smokers by 590,000 and would save an estimated 340,000 lives [43].

We conducted a sensitivity analysis to quantify the impact of variations in key inputs. With regard to the change in price elasticities depending on the price level, we made the assumption that elasticity is constant across the entire range of prices; as it is common in the respective literature [44]. Estimates of the price elasticity of demand for tobacco products are robust. Studies have shown that the price elasticity varied very narrowly from − 0.2 to − 0.8, clustering around − 0.5 in low and middle-income countries, implying that variations of price elasticities are not stark irrespective of what the price level is [45]. The sensitivity analysis illustrates if the value of the tax increase were 25, 50, or 100% instead of 75%, the distributional consequences would even out. We explore how switching to cheaper cigarette brands may affect the consequences in terms of health and financial gains. For instance, the premature deaths averted would decrease by 75% by assuming that 75% of consumers switched. Apart from additional tax revenues, higher switching could reduce considerably the examined consequences more. In order to reduced uncertainty, a sensitivity analysis was also performed to assess how the choice of different poverty thresholds affect (averted) impoverishment cases. We noticed that increasing the poverty line from US$ 1.9 to US$ 5.5 per day would enhance slightly the policy progressiveness. Our study has several limitations. First, due to the lack of clear evidence for all the MPOWER measures, our analysis only focused on raising taxation. Further work is needed to clarify the impact of alternative policies included in MPOWER and develop models that can estimate the association between tobacco taxation and lost productivity resulting from tobacco-related death and disease. Second, we assumed that no smoker will not quit smoking if an increasing price intervention is not enforced. This may overestimate the potential impact of the price policy. Third, we don’t have data on variations in the prevalence of smoking-related diseases, and distribution of age groups among quintiles. Fourth, data shortcomings preclude us from seeing the effect of various cigarette smoke inhalation patterns, type of cigarette and history of smoking on the outcomes under consideration. Stratified economic and epidemiological data are needed to quantify the effect of such variations. Fifth, we only modeled the consequences of cigarette excise tax fully passed onto smokers. The chance of succeeding in the implementation of tobacco taxation requires attention to the role of predominant stakeholders and the Iranian tobacco tax structure. It should be noted that owing to the scarcity of data our estimations do not include the impact of cigarette price increase through taxation on other tobacco products (cross-price elasticities). Therefore, effective price policy requires to estimate cross-price effects and understand the differences in price elasticity of demand among them. Furthermore, the estimates for financial protection are conservative and should be interpreted with great caution considering that they do not include costs related to tobacco-related disease morbidity. Lastly, our model did not take into account health gains from a reduction in the intensity of smoking as well as from preventing secondhand smoke.

## Conclusion

It is often of interest for policy reasons to appreciate the potential aspects of program interventions in many spheres of public policy, especially in health. ECEA describes a correct approach in this area, since it provides an option for decision makers and implementers to examine far-reaching effects compared to the conventional cost-effectiveness analysis. By applying ECEA, cigarette excise tax could be analyzed in great detail and by expenditure quintile by exploring the health and financial implications of tobacco control efforts. Regarding the low share of taxes in retail prices and relative inelasticity of demand for the cigarette, Iran has ample room to increase its excises. Designing a range of evidence-based price policies towards reducing tobacco consumption necessitates considering various elements including inflation, affordability of tobacco, tax structure and administration, employment, as well as an understanding of key determinants of tobacco demand to attain health and revenue objectives. The results revealed that increased cigarette tax can be pro-poor in terms of OOP savings and financial risk protection. In summary, this policy is progressive while the major part of financial benefits concentrated among wealthier subgroups and could help the society meet sustainable development goals.

## Supplementary Information

**Additional file 1.**

## Data Availability

The datasets used and/or analyzed during the current study are available from the corresponding author on reasonable request.
