# Supplementary material for: Distributional health and financial consequences of increased cigarette tax in Iran: extended cost-effectiveness analysis
Source: Health Econ Rev. 2021 Aug 13;11:30. doi: 10.1186/s13561-021-00328-w (PMC8364107; doi:10.1186/s13561-021-00328-w)
Supplement: Supplementary file 1 — Additional file 1. [file 13561_2021_328_MOESM1_ESM.docx]

**Supplementary document**

**Appendix 1: Technical Data Appendix**

**Appendix 2: Two-Part Model Regressions**

**Appendix 3: Quintile-specific Elasticities**

**Appendix 4: Premature Deaths Averted**

**Appendix 5: Years of Life Gained**

**Appendix 6: Additional Tax Revenues**

**Appendix 7: Change in Household Expenditures on Cigarette**

**Appendix 8: Averted Expenditures on Smoking-related Diseases**

**Appendix 9: Financial Risk Protection**
**Appendix 10: Sensitivity Analysis**

**Appendix 1: Technical Data Appendix**

Although there are some shortcomings in measuring consumption expenditures, they are less volatile than measuring household income, especially in less-developed countries. Thus, consumption expenditures compared to income can better reflect the economic status of households. Therefore, the use of expenses as a suitable proxy for income in the literature is generally accepted (1). In this study, first, total household expenditure was calculated by the sum of expenses related to different commodity groups and then translated into per adult equivalent by dividing household expenditure by the square root of household size which is known as the “Square root scale” (2).

**Table 1:Descriptive statistics of sample households and cigarette use (Means)**

| Household socio-economic status | | | | |  |
| --- | --- | --- | --- | --- | --- |
| **Fifth(richest) quintile** | **Fourth quintile** | **Third**  **quintile** | **Second quintile** | **First (poorest) quintile** |  |
| 110,783 | 110,666 | 110,540 | 110,299 | 108,518 | Sample size(household) |
| 26,200,000  (15,700,000) | 12,700,000  (1,460,300) | 8,968,216  (839,906) | 6,394,827  (684,814) | 3,729,587  (1,032,995) | Monthly average expenditure per equivalent adult (IR Rial) |
| 1525.7 (1248.8) | 1292.1 (810) | 1145.6 (622) | 1068.2 (971) | 935.7 (421) | Cigarette price (per stick, IR Rial) |
| 2.4 (2.2) | 3 (2.4) | 3.5 (2.5) | 4.3 (3.2) | 5.1 (4.1) | Household expenditures spent on cigarettes (%) |
| 27  (18) | 27  (19) | 28  (19) | 28  (19) | 28  (19) | Percentage of households with men aged between 19 and 64 (%) |
| 33  (17) | 32  (17) | 31  (17) | 30  (18) | 27  (22) | Percentage of households with women aged between 19 and 64 (%) |
| 503 (330) | 509 (341) | 508 (319) | 503 (308) | 507 (328) | Number of cigarettes use per month |
| 25  (29) | 16  (23) | 10  (19) | 7  (16) | 4  (12) | Percentage of households with at least one member who holds university education (%) |
| 55  (31) | 59  (30) | 59  (30) | 56  (31) | 44  (33) | Percentage of households with primary education (%) |
| 13  (24) | 13  (23) | 13  (23) | 15  (25) | 24  (33) | Proportion of unemployed household members (%) |
| 11.1 (4.9) | 11.2 (5.1) | 11.3 (5.2) | 11.2 (5.2) | 10.9 (4.9) | Unemployment rate (%) |
| 15.2 (8.3) | 15.4 (8.1) | 15.4 (8) | 15.2 (7.7) | 13.9 (7.3) | Divorce rate (%) |
| 66 (47) | 57 (49) | 49 (50) | 40 (49) | 27 (44) | Residence Area (urbanity %) |

Note: Source is 2002-2017 HIES, Standard Deviation is represented in parenthesis

**Appendix 2: Two-Part Model Regressions**

This is a two-stage approach, the first stage is a probit model to address individual’s participation decisions (a dichotomous choice model), and the second one employed an ordinary least squares (OLS) regression to examine the quantity of demand (a contribution level model) on condition that the choice was made in the first stage (3). In the first stage, the dependent variable is a dummy equal to 1 if the individuals' observation includes a contribution to an activity of interest, and 0 otherwise. So, the first stage attempts to determine the factors that explain the decision of whether or not to contribute while stage 2 is limited to those consumers having contribution. The two-part model can be expressed as follows:

parametric binary probability model (part one) (1)

$$Z_{i}^{*}=W_{i}^{'}\mathcal{a+}e_{i}$$

$$Z_{i}=0 if Z_{i}^{*}\leq0$$

$$Z_{i}=1 if Z_{i}^{*} >0$$

$y_{i}^{*}=X_{i}^{´}\beta+u_{i}$

Linear model (part two) (2)

$$y_{i}=y_{i}^{*} if Z_{i}=1$$

$$y_{i} not observed Z_{i}=0$$

Where, *z_i_* is a dichotomous variable which detects whether or not y is observed, *y_i_* being observed only when *z_i_* = 1, *W_i_* denotes observable features including the overlapping variables with *X_i_*, and *α* denotes the vectors of parameters to be estimated.

In the linear model, *y_i_* denotes the dependent variable, *X_i_* denotes the observable features of the independent variables, *β* is the parameters to be estimated and *µ_i_* is a normally distributed error term with a mean of zero and a standard deviation σ to be estimated (4).

**Appendix 3: Quintile-specific Elasticities**

This appendix includes the results of the 2-part model regressions. The participation elasticities are calculated using coefficients from the probit model with the following variable definitions:

- *Smoke* Dummy variable that takes a value of 1 if the household spent any money on cigarettes;
- *Price* A continuous variable representing the price of cigarette (in natural logarithm);
- *Income* Total monthly household income of employed family members from self-employment and salary by Iranian rials (in natural logarithm);
- *Divorce rate* A province-level variable representing the total divorce rate of the province where household residing in;
- *Unemployment rate* A province-level variable representing the total unemployment rate of the province where household residing in;
- *share of members aged 15 to 18* Proportion of household members at the age between 15 and 18 to household size;
- *share of jobless members* Proportion of unemployed household members aged between15 and 64 to household size;
- *share of primary educated* A variable showing proportion of household with at least one member who had primary education;
- *share of university educated* share of household with at least one member who holds a university degree;
- *Age* Age of the household head;
- *Sex* Dummy variable that takes a value of 1 if the head of household is male
- *Year* Sixteen dummy variables, from 2002 to 2017, that each takes a value of 1 if the household was interviewed in the corresponding year (2002 is the omitted (reference) year in the regression equations).

In the second part using ordinary least squares regression, we adopted a linear model to estimate the conditional price and income elasticities. Specifically, we estimated;

$Ln\left( Consumption \right)= \beta0 + \beta1ln\left( income \right)+ \beta2 Ln\left( Price \right)+ \beta3\left( Divorce rate \right)+ \beta4\left( Unemployment rate \right)+ \beta5\left( Education \right)+\beta6\left( Year \right)+ℇ$ (3)

| Table2. Estimated probit regression model on smoking participation among each expenditure quintile, based on 2002-2017 Households Income and Expenditure Survey data | | | | | | | | | | | | |
| --- | --- | --- | --- | --- | --- | --- | --- | --- | --- | --- | --- | --- |
| Q1 (n=108,518)  Q2 (n= 110,299)  Q3 (n= 110,540)  Q4 (n= 110,666)  Q5 (n= 110,783)  Independent variables | | |  | |  | |  | |  | |  | |
| Income | 0.035 (0.005) ^***^ | | - 0.037 (0.005) ^***^ | | - 0.029 (0.005) ^***^ | | - 0.046 (0.005) ^***^ | | - 0.015 (0.005) ^***^ | |  |  |
| Price | - 0.089 (0.011) ^***^ | | - 0.084 (0.011) ^***^ | | - 0.098 (0.011) ^***^ | | - 0.093 (0.012) ^***^ | | - 0.099 (0.013) ^***^ | |  |  |
| Divorce rate | 0.08 (0.011) ^***^ | | 0.033 (0.010) ^***^ | | 0.025 (0.010) ^*^ | | 0.038 (0.009) ^***^ | | 0.015 (0.008) | |  |  |
| Unemployment rate | 0.012 (0.001) ^***^ | | 0.015 (0.000) ^***^ | | 0.010 (0.001) ^***^ | | 0.009 (0.001) ^*^ | | 0.007 (0.000) | |  |  |
| Share of members aged 15 to 18 | - 0.155 (0.039) ^***^ | | - 0.054 (0.036) | | - 0.010 (0.036) | | 0.167 (0.036) ^***^ | | 0.270 (0.036) ^***^ | |  |  |
| Share of members aged>64 | - 0.155 (0.034) ^***^ | | - 0.511 (0.034) ^***^ | | - 0.625 (0.035) ^***^ | | - 0.595 (0.037) ^***^ | | - 0.617 (0.037) ^***^ | |  |  |
| Share of male members aged 19 to 64 | 0.528 (0.034) ^***^ | | 0.430 (0.029) ^***^ | | 0.412 (0.029) ^***^ | | 0.465 (0.029) ^***^ | | 0.482 (0.029) ^***^ | |  |  |
| Share of female members aged 19 to 64 | - 0.195 (0.034) ^***^ | | -0.375 (0.032) ^***^ | | - 0.341 (0.032) ^***^ | | - 0.255 (0.032) ^***^ | | - 0.152 (0.033) ^***^ | |  |  |
| Share of jobless member | - 0.122 (0.024) ^***^ | | - 0.130 (0.024) ^***^ | | - 0.078 (0.025) ^**^ | | - 0.026 (0.024) | | 0.046 (0.023) | |  |  |
| Share of primary educated | 0.489 (0.019) ^***^ | | 0.253 (0.019) ^***^ | | 0.141 (0.019) ^***^ | | 0.148 (0.019) ^***^ | | 0.036 (0.020) | |  |  |
| Share of university educated | - 0.251 (0.043) ^***^ | | - 0.588 (0.033) ^***^ | | - 0.788 (0.029) ^***^ | | - 0.785 (0.026) ^***^ | | - 0.743 (0.023) ^***^ | |  |  |
| Age | 0.001 (0.000) ^***^ | | 0.005 (0.000) ^***^ | | 0.007 (0.000) ^***^ | | 0.008 (0.000) ^***^ | | 0.007 (0.000) ^***^ | |  |  |
| Sex | - 0.746 (0.019) ^***^ | | - 0.694 (0.020) ^***^ | | - 0.677 (0.021) ^***^ | | - 0.641 (0.022) ^***^ | | - 0.555 (0.022) ^***^ | |  |  |
| Year |  | |  | |  | |  | |  | |  |  |
| 1 (2003) | 0.111 (0.026) ^***^ | | 0.157 (0.026) ^***^ | | 0.125 (0.026) ^***^ | | 0.103 (0.027) ^***^ | | 0.059 (0.027) ^*^ | |  |  |
| 2 | 0.119 (0.030) ^***^ | | 0.284 (0.028) ^***^ | | 0.152 (0.028) ^***^ | | 0.096 (0.028) ^***^ | | 0.073 (0.028) ^*^ | |  |  |
| 3 | 0.157 (0.029) ^***^ | | 0.180 (0.027) ^***^ | | 0.126 (0.026) ^***^ | | 0.099 (0.025) ^***^ | | 0.084 (0.024) ^**^ | |  |  |
| 4 | 0.109 (0.027) ^***^ | | 0.168 (0.026) ^***^ | | 0.128 (0.025) ^***^ | | 0.085 (0.024) ^***^ | | 0.044 (0.023) | |  |  |
| 5 | 0.041 (0.025) | | 0.037 (0.024) | | - 0.042 (0.024) | | - 0.086 (0.023) ^***^ | | - 0.098 (0.022) ^***^ | |  |  |
| 6 | 0.019 (0.025) | | 0.019 (0.024) | | - 0.047 (0.023) ^*^ | | - 0.060 (0.023) ^**^ | | - 0.072 (0.021) ^**^ | |  |  |
| 7 | - 0.058 (0.019) ^*^ | | - 0.026 (0.022) | | - 0.054 (0.021) ^*^ | | - 0.063 (0.021) ^**^ | | - 0.096 (0.021) ^***^ | |  |  |
| 8 | - 0.090 (0.022) ^***^ | | -0.056 (0.021) ^*^ | | -0.072 (0.021) ^*^ | | -0.054 (0.021) ^*^ | | -0.115 (0.021) ^***^ | |  |  |
| 9 | - 0.107 (0.023) ^***^ | | - 0.084 (0.021) ^***^ | | - 0.112 (0.021) ^***^ | | - 0.122 (0.021) ^***^ | | - 0.134 (0.021) ^***^ | |  |  |
| 10 | - 0.231 (0.025) ^***^ | | - 0.257 (0.022) ^***^ | | - 0.209 (0.021) ^***^ | | - 0.216 (0.022) ^***^ | | - 0.210 (0.023) ^***^ | |  |  |
| 11 | - 0.037 (0.024) | | - 0.081 (0.020) ^***^ | | - 0.078 (0.021) ^***^ | | - 0.039 (0.021) | | - 0.047 (0.023) ^*^ | |  |  |
| 12 | - 0.004 (0.026) | | - 0.012 (0.021) | | - 0.023 (0.022) | | - 0.013 (0.023) | | - 0.025 (0.027) | |  |  |
| 13 | 0.033 (0.024) | | 0.127 (0.022) ^***^ | | 0.133 (0.022) ^***^ | | 0.196 (0.024) ^***^ | | 0.160 (0.027) ^***^ | |  |  |
| 14 | - 0.052 (0.023) ^*^ | | 0.013 (0.021) | | - 0.008 (0.022) | | - 0.012 (0.024) | | - 0.021 (0.029) | |  |  |
| 15 (2017) | - 0.104 (0.024) ^***^ | | -0.037 (0.022) | | -0.014 (0.023) | | -0.025 (0.025) | | -0.002 (0.029) | |  |  |
| Constant ($\boldsymbol{\alpha}_{\mathbf{0}}$) | -0.353 (0.114) ^**^ | | 0.946 (0.114) ^***^ | | 1.11 (0.117) ^***^ | | 1.38 (0.119) ^***^ | | 0.988 (0.121) ^***^ | |  |  |

Values are presented as a coefficient (standard error). ^*^p<0.05, ^**^p<0.01, ^***^p<0.001.

The following equation was applied to estimate the price elasticity of participation (EP)(5):

$EP=\frac{\beta_{i}}{\sqrt{2\pi}}exp\left[ -\frac{1}{2}({\betaˊ\bar{X})}^{2} \right]\frac{1}{E(YІX)}$ (4)

| Table 3. Estimated Linear regression model on smoking intensity among each expenditure quintile, based on 2002-2017 Households Income and Expenditure Survey data | | | | | | |  |
| --- | --- | --- | --- | --- | --- | --- | --- |
| Q1 (n= 17,148)  Q2 (n= 24,778)  Q3 (n= 26,152)  Q4 (n= 25,722)  Q5 (n= 24,690)  Independent variables |  |  |  |  |  | | |
| Income | 0.0472 (0.006) ^**^ | -0.000 (0.005)^**^ | 0.007 (0.006) ^**^ | 0.019 (0.006) ^* *^ | | 0.028 (0.006) ^**^ | |
| Price | - 0.404 (0.013) ^***^ | - 0.369 (0.010) ^***^ | - 0.366 (0.010) ^***^ | - 0.379 (0.010) ^***^ | | - 0.323 (0.010) ^***^ | |
| Divorce rate | - 0.005(0.013) ^***^ | - 0.020 (0.010) ^***^ | - 0.005 (0.010) ^***^ | - 0.006 (0.009) ^**^ | | - 0.007 (0.009) ^**^ | |
| Unemployment rate | 0.004 (0.001) ^**^ | 0.006 (0.001) ^**^ | 0.005 (0.001) ^**^ | 0.006 (0.001) ^**^ | | 0.004 (0.001) ^**^ | |
| Education  (Ref: no education) |  |  |  |  | |  | |
| Elementary school | - 0.024 (0.013) ^***^ | -0.045 (0.010) ^***^ | - 0.053 (0.011) ^***^ | - 0.072(0.013) ^***^ | | - 0.070 (0.015) ^***^ | |
| Junior high school | - 0.066 (0.016) ^***^ | - 0.119 (0.013) ^***^ | -0.165 (0.013) ^***^ | - 0.185 (0.015) ^***^ | | - 0.195 (0.017) ^***^ | |
| Senior high school | -0.148 (0.038) ^***^ | - 0.260 (0.025) ^***^ | - 0.267 (0.021) ^***^ | - 0.302 (0.021) ^***^ | | - 0.302 (0.019) ^***^ | |
| Diploma | - 0.257 (0.041) ^***^ | - 0.315 (0.027) ^***^ | - 0.297 (0.023) ^***^ | - 0.373 (0.022) ^***^ | | - 0.302 (0.022) ^***^ | |
| Bachelor | - 0.374 (0.100) | -0.367 (0.054) | - 0.448 (0.037) ^***^ | - 0.495 (0.027) ^***^ | | - 0.494 (0.021) ^***^ | |
| Master and above | - 0.728 (0.385) | - 0.545 (0.185) | - 0.518 (0.092) | - 0.561 (0.069) | | - 0.487 (0.037) ^***^ | |
| Year |  |  |  |  | |  | |
| 1 (2003) | - 0.023 (0.031) ^***^ | - 0.046 (0.028) ^***^ | - 0.075 (0.029) ^***^ | - 0.088 (0.031) ^***^ | | - 0.050 (0.032) ^***^ | |
| 2 | - 0.046 (0.035) ^***^ | 0.025 (0.030) ^***^ | - 0.057 (0.030) ^***^ | - 0.076 (0.033) ^***^ | | - 0.039 (0.034) ^***^ | |
| 3 | 0.340 (0.034) ^***^ | 0.233 (0.029) ^***^ | 0.171 (0.029) ^***^ | 0.1061 (0.030) ^***^ | | 0.095 (0.030) ^***^ | |
| 4 | - 0.024 (0.033) ^***^ | - 0.045 (0.028) ^***^ | - 0.082 (0.028) ^***^ | - 0.123 (0.029) ^***^ | | - 0.082 (0.029) ^***^ | |
| 5 | - 0.004 (0.031) ^***^ | - 0.008 (0.027) ^***^ | - 0.006 (0.028) ^***^ | - 0.047 (0.029) ^***^ | | - 0.053 (0.029) ^***^ | |
| 6 | - 0.015 (0.032) ^***^ | 0.069 (0.028) ^***^ | 0.015 (0.028) ^***^ | 0.025 (0.029) ^***^ | | 0.007 (0.029) ^***^ | |
| 7 | - 0.019 (0.031) ^***^ | - 0.033 (0.027) ^***^ | - 0.105 (0.027) ^***^ | - 0.129 (0.029) ^***^ | | - 0.027 (0.030) ^***^ | |
| 8 | - 0.078 (0.031) ^***^ | - 0.064 (0.027) ^***^ | - 0.081 (0.027) ^***^ | - 0.121 (0.029) ^***^ | | - 0.054 (0.030) ^***^ | |
| 9 | -0.057 (0.032) ^***^ | - 0.004 (0.027) ^***^ | - 0.018 (0.027) ^***^ | - 0.041 (0.029) ^***^ | | - 0.038 (0.030) ^***^ | |
| 10 | 0.005 (0.034) ^***^ | - 0.011 (0.028) ^***^ | - 0.037 (0.028) ^***^ | - 0.038 (0.030) ^***^ | | - 0.030 (0.032) ^***^ | |
| 11 | - 0.046 (0.033) ^***^ | - 0.018 (0.027) ^***^ | - 0.052 (0.027) ^***^ | - 0.041 (0.029) ^***^ | | - 0.019 (0.032) ^***^ | |
| 12 | 0.041 (0.032) ^***^ | 0.003 (0.027) ^***^ | - 0.003 (0.027) ^***^ | - 0.025 (0.031) ^***^ | | - 0.039 (0.035) ^***^ | |
| 13 | 0.115 (0.031) ^***^ | 0.111 (0.027) ^***^ | 0.052 (0.028) ^***^ | 0.000 (0.031) ^***^ | | 0.044 (0.036) ^***^ | |
| 14 | 0.113 (0.031) ^***^ | 0.090 (0.027) ^***^ | 0.070 (0.028) ^***^ | 0.029 (0.032) ^***^ | | 0.042 (0.038) ^***^ | |
| 15 (2017) | 0.044 (0.031) ^***^ | 0.042 (0.027) ^***^ | 0.027 (0.028) ^***^ | 0.019 (0.032) ^***^ | | - 0.013 (0.037) ^***^ | |
| Constant (β0) | 7.848 (0.134) | 8.481 (0.117) | 8.458 (0.119) | 8.421 (0.126) | | 7.937 (0.124) | |

Values are presented as coefficient (standard error). Ref, reference

**Table 4. Price elasticities by expenditure quintile**

| **Elasticities** | **Poorest** | **Second** | **Third** | **Fourth** | **Richest** |
| --- | --- | --- | --- | --- | --- |
| *Participation* | - 0.07 | - 0.11 | - 0.12 | - 0.12 | - 0.11 |
| *Consumption* | - 0.40 | - 0.36 | - 0.36 | - 0.37 | - 0.32 |
| *Total* | - 0.47 | - 0.47 | - 0.48 | - 0.49 | - 0.43 |

The total price elasticity was computed by summing the elasticities from the first and the second part of the estimation.

**Appendix 4: Premature Deaths Averted**

The number of smokers quitting is dependent on the participation elasticity. following the price hike, the number of smokers is calculated using the formula below:

$S_{post,q}=\left( 1+\varepsilon_{p,q}\Delta P \right)S_{ante,q}$ (5)

where $S_{ante,q}$ is the number of smokers in the expenditure quintile 𝑞 before the price hike, and Δ𝑃 is the relative change in the retail price of cigarettes (here 75% of the final price). Accordingly, the number of premature deaths averted could be:

$D_{a,q}=(S_{ante,q}- S_{post,q})\delta{RR}_{a}$ (6)

where 𝛿 denotes the probability that a continuing smoker will die early (0.50 according to Doll et al. (6)) and 𝑅𝑅 𝑎 is the relative risk reduction of premature death depending on age at quitting 𝑎. Based on the age-specific relative risk reductions modeled by Verguet et al. (7) in Table 5, the number of premature deaths averted was estimated.

**Table 5. Reduction of smoking-attributable death risk by age at quitting**

| **Age-groups** | $\boldsymbol{RR}_{\boldsymbol{a}}$ |
| --- | --- |
| • 15–19 years–old: | 96.9% |
| • 20–24 years–old: | 94.8% |
| • 25–29 years–old: | 92.1% |
| • 30–34 years–old: | 89.2% |
| • 35–39 years–old: | 86.6% |
| • 40–44 years–old: | 83.7% |
| • 45–49 years–old: | 79.5% |
| • 50–54 years–old: | 72.9% |
| • 55–59 years–old: | 62.8% |
| • 60–64 years–old: | 49.9% |
| • 65–69 years–old: | 36.4% |
| • 70–74 years–old: | 24.7% |
| • 75–79 years–old: | 15.7% |
| • ≥80 years–old: | 9.1% |

**Appendix 5: Years of Life Gained**

Over the succeeding 60 years and in male cigarette quitters, years of life gained after increased cigarette price by 75% of retail price through taxation were estimated. We hypothesized that all the subjects would die when they reached the age of 75 years representing life expectancy at birth for men in Iran. Therefore, for each age group, years of life gained were estimated by fitting a linear regression model of the log of the difference between life expectancy at birth (here 75 years) and age at quitting. The fitted model has been employed in similar published studies (8) in the following way:

$YLG(Quitter)=-10.3+5.5\ln(LE-age)\text{ for}\text{ }\text{ }LE-age\geq7$ $YLG(Quitter)=0\text{ otherwise}$ $YLG(Non-quitter)=0$ (7)

where *LE* and *age* denote life expectancy at birth and the age at quitting respectively. the results of our estimation by quintile have been shown in the table below.

**Table 6. Years of Life Gained following a 75% increase in cigarette price**

| Quintile | I (poorest) | II | III | IV | V (Richest) | Total |
| --- | --- | --- | --- | --- | --- | --- |
| YLG | 205,869(11%) | 392,289(21%) | 444,837(24%) | 446,292(24%) | 373,293(20%) | 1,862,580 |

**Appendix 6: Additional Tax Revenues**

Following the increase in the price of cigarettes and the decrease in demand for it, tax revenues can be calculated by expenditure quintile using the following equation:

$R_{q}=\sum_{a} S_{a,q}Cigs[{tax}_{2}(1+\varepsilon_{q,a}\frac{\Delta P}{P})- {tax}_{1}]$ (8)

where $S_{a,q}$ is the number of smokers in the age group 𝑎 in the quintile q in advance of price increase, 𝐶𝑖𝑔𝑠 is the number of packs of cigarettes smoked per smoker yearly, $\frac{\Delta P}{P}$ is the relative change in the retail price of cigarettes (here 75% of the final price), $\varepsilon_{q,a}$ is the total price elasticity in the age group 𝑎 in the quintile q, and${tax}_{1}$ and ${tax}_{2}$ are the excise tax revenues yielded per cigarette pack before- and after-tax rise respectively (9).

**Appendix 7: Change in Household Expenditures on Cigarette**

The net change in expenditures on cigarettes following the price increase in the quintile q could be defined as follows:

$T_{q}=\sum_{a} S_{a,q}Cigs[P_{2}(1+\varepsilon_{q,a}\frac{\Delta P}{P})- P_{1}]$ (9)

where $P_{1}$ is the pack price before price increase, and $P_{2}$ is the pack price after the price increase. Other quantities are the same as in Appendix 6.

**Appendix 8: Averted Expenditures on Smoking-related Diseases**

After estimating the premature deaths averted based on the findings of the previous work in this field, we distributed these premature deaths into four smoking-related diseases including ischemic heart disease, lung cancer, stroke, and chronic obstructive pulmonary disease. Lastly, depending on the causes of death and share of health care utilization rate for the aforementioned diseases per quintile, treatment-related costs averted in quintile q were calculated by:

${TC}_{q}=\left( \sum_{a} D_{q}\sum_{d} p_{d}\upsilon_{d,q}C_{d} \right)$ (10)

where $D_{q}$ represents the number of premature deaths averted in the quintile q, $p_{d}$ is the share of disease 𝑑 to smoking-related premature deaths, $C_{d}$ is the treatment cost of disease 𝑑, and $\upsilon_{d,q}$is healthcare utilization for disease 𝑑 in quintile q. Therefore, Out-of- Packet expenditures averted would account for 41% of ${TC}_{q}$ (Table 1 in the main text) (9).

**Appendix 9: Financial Risk Protection**

we estimated the number of averted cases of impoverishment and averted catastrophic out-of-pocket expenditures due to the lower incidence of tobacco-related diseases and resulting averted OOP costs. To do so, we counted the number of individuals on whom OOP expenditures would fall, which is equivalent to $\sum_{a} D_{q}\sum_{d} p_{d}\upsilon_{d,q}$individuals. Among them, we counted the number of individuals for whom the size of OOP expenditures would push them under the poverty line threshold 𝑃Y. Monthly data on expenditure per adult equivalent as a proxy for income can be characterized by a probability distribution function. In our analysis, the 2-parameter gamma distribution is fitted for the data, as used in the literature (10). The data included a 16-year period from 2002 to 2017. we converted the total expenditure into per adult equivalent and adjusted it by the general consumer price index in 2017. Then, according to the poverty line threshold, the percentage of the population below the poverty line was determined. We applied a poverty line threshold PL of US$3.20 per day (or US$1169 yearly). A poverty case was counted when first individual expenditure per adult equivalent was above the poverty line (𝑦 > PL) and second individual expenditure per adult equivalent minus OOP expenditures was below the poverty line (𝑦 − 𝑐q < PL). Similarly, we estimated the number of cases of averted catastrophic expenditures attributed to 𝑂𝑂𝑃 costs. Among the individuals for whom OOP would be incurred, we counted the number of individuals that their OOP costs would exceed 20% of their annual expenditure per adult equivalent.

**Appendix 10: Sensitivity Analysis**

Three different univariate sensitivity analyses were conducted to assess the robustness of our finding relative to changes in key parameters. First, we tested the outcomes by applying a varying increase in the retail price of cigarettes to the quintiles in two additional scenarios. The results are shown in Table 7 below.

| Table 7. retail price increase is set at 25% | | | | | | |
| --- | --- | --- | --- | --- | --- | --- |
| Quintiles | I(poorest) | II | III | IV | V(richest) | Total |
| Premature deaths averted | 7,186(11%) | 13,941(21%) | 15,882(24%) | 15,836(24%) | 13,324(20%) | 66,169 |
| Years of life gained | 68,623(11%) | 130,763(21%) | 148,279(24%) | 148,764(24%) | 124,431(20%) | 620,860 |
| Expenditures on tobacco-related disease treatment averted (2017; in $US) | 5,860,690  (9%) | 12,913,925  (20%) | 15,991,223  (24%) | 16,104,356  (25%) | 14,623,038  (22%) | 65,493,232 |
| Total OOP savings  (2017; in $US) | 2,402,883  (9%) | 5,294,709  (20%) | 6,556,401  (24%) | 6,602,786  (25%) | 5,995,446  (22%) | 26,852,225 |
| Additional tax revenues from excise tax (2017; in $US) | 56,066,900  (12%) | 76,970,138  (17%) | 109,304,322  (24%) | 117,570,705  (26%) | 94,139,987  (21%) | 454,052,052 |
| Change in annual expenditures on cigarette (2017; in $US)  % of individual income | 26,206,908  (1.51%) | 35,977,543  (1.13%) | 49,683,783  (1.09%) | 51,918,687  (0.77%) | 48,784,027  (0.55%) | 216,007,944 |
| Poverty cases averted | 1,806(0.17%) | 3,942(0.30%) | 0 | 0 | 0 | 5,748(0.09%) |
| No. of cases of catastrophic expenditure averted | 4,304(0.41%) | 9,484(0.72%) | 4,932(0.36%) | 0 | 0 | 18,820(0.29%) |
| retail price increase is set at 100% | |  |  |  |  |  |
| Premature deaths averted | 28,744(11%) | 55,764(21%) | 63,528(24%) | 63,344(24%) | 53,296(20%) | 264,676 |
| Years of life gained | 274,492  (11%) | 523,052  (21%) | 593,116  (24%) | 595,056  (24%) | 497,724  (20%) | 2,483,440 |
| Expenditures on tobacco-related disease treatment averted (2017; in $US) | 23,442,760  (9%) | 51,655,700  (20%) | 63,964,892  (24%) | 64,417,424  (25%) | 58,492,152  (22%) | 261,972,928 |
| Total OOP savings  (2017; in $US) | 9,611,532  (9%) | 21,178,836  (20%) | 26,225,604  (24%) | 26,411,144  (25%) | 23,981,784  (22%) | 107,408,900 |
| Additional tax revenues from excise tax (2017; in $US) | 134,687,625  (12%) | 184,902,768  (17%) | 258,355,670  (24%) | 273,326,766  (25%) | 240,492,069  (22%) | 1,091,764,897 |
| Change in annual expenditures on cigarette (2017; in $US)  % of individual income | 15,247,656  (0.87%) | 20,932,389  (0.66%) | 19,873,513  (0.43%) | 10,718,697  (0.16%) | 59,068,227  (0.67%) | 125,840,482 |
| Poverty cases averted | 7,224 (0.69%) | 15,769(1.21%) | 0 | 0 | 0 | 22,993 |
| No. of cases of catastrophic expenditure averted | 17,216  (1.65%) | 37,936  (2.91%) | 19,730  (1.46%) | 0 | 0 | 74,882 |

Second, we checked the sensitivity of our findings by incorporating a parameter, which indicates the proportion of smokers who demand low-priced cigarettes instead of reducing or quitting. In this case, the number of smokers following retail price increase in expenditure quintile 𝑞 is calculated using the equation below:

$S_{post,q}=(1+\varepsilon_{p,q}\Delta P\left( 1-S_{w} \right))S_{ante,q}$ (11)

Where $S_{ante,q}$ and $S_{post,q}$ denote the number of smokers in quintile q before and after price increase respectively. $\varepsilon_{p,q}$ is participation elasticity in quintile q, ∆𝑃 is the relative change in the price of cigarettes, and $S_{w}$ represents the proportions of smokers who switch. Thus, the change in tax revenues by considering switchers in quintile 𝑞 would become:

$R_{q}=\sum_{a} S_{ante,q}Cigs[{tax}_{2}(1+\varepsilon_{q}\Delta P(1-S_{w}))- {tax}_{1}]$ (12)

Where 𝐶𝑖𝑔 is the number of cigarette packs smoked per smoker per year, $\varepsilon_{q}$ is total price elasticity, and other variables are the same as in Appendix 6. The results are presented and compared with the base case scenario in the Figures (main text).

Third, we employed two alternative poverty thresholds to quantify the financial risk protection benefitted to all expenditure quintiles. Table 8 below gathers the results of changes in the poverty lines used in the model.

**Table 8. Impact of a 75% increase in the retail price of cigarette on financial risk protections under 3 poverty thresholds**

|  |  | | | | | |
| --- | --- | --- | --- | --- | --- | --- |
|  | US$1.90 | | US$3.20 | | US$5.5 | |
| Quintiles | Poverty cases  averted | Catastrophic  cases  averted | Poverty cases  averted | Catastrophic  cases  averted | Poverty cases  averted | Catastrophic  cases  averted |
| I(poorest) | 8,053(0.77%) | 12,912(1.23%) | 5,418(0.52%) | 12,912(1.23%) | 0 | 12,912(1.23%) |
| II | 0 | 7,113 (0.54%) | 11,827(0.90%) | 28,452(2.18%) | 16,824(1.29%) | 28,452(2.18%) |
| III | 0 | 0 | 0 | 14,797(1.10%) | 9,009(0.66%) | 35,232(2.62%) |
| IV | 0 | 0 | 0 | 0 | 0 | 35,481(2.65%) |
| V(richest) | 0 | 0 | 0 | 0 | 0 | 6,120(0.48%) |
| Total | 8,053(0.12%) | 20,025 (0.31%) | 17,245(0.27%) | 56,161 (0.89%) | 25,833(0.81%) | 118,197 (1.88%) |

**References**

1. Rutstein SO, Johnson K. The DHS wealth index. DHS comparative reports no. 6. Calverton: ORC Macro. 2004.

2. Scales OWAE. OECD Project on Income Distribution and Poverty.

3. Mihaylova B, Briggs A, O'Hagan A, Thompson SG. Review of statistical methods for analysing healthcare resources and costs. Health economics. 2011;20(8):897-916.

4. Mullahy J. Much ado about two: reconsidering retransformation and the two-part model in health econometrics. Journal of health economics. 1998;17(3):247-81.

5. Wooldridge JM. Econometric analysis of cross section and panel data MIT press. Cambridge, MA. 2002;108.

6. Doll R, Peto R, Boreham J, Sutherland I. Mortality in relation to smoking: 50 years' observations on male British doctors. Bmj. 2004;328(7455):1519.

7. Verguet S, Tarr G, Gauvreau CL, Mishra S, Jha P, Liu L, et al. Distributional benefits of tobacco tax and smoke–free workplaces in China: A modeling study. Journal of global health. 2017;7(2).

8. Salti N, Brouwer E, Verguet S. The health, financial and distributional consequences of increases in the tobacco excise tax among smokers in Lebanon. Social Science & Medicine. 2016;170:161-9.

9. Verguet S, Gauvreau CL, Mishra S, MacLennan M, Murphy SM, Brouwer ED, et al. The consequences of tobacco tax on household health and finances in rich and poor smokers in China: an extended cost-effectiveness analysis. Economics of Tobacco Control in China: From Policy Research to Practice: World Scientific; 2016. p. 215-43.

10. Salem AB, Mount TD. A convenient descriptive model of income distribution: the gamma density. Econometrica: journal of the Econometric Society. 1974:1115-27.
